# Some things get better with age: differences in salicylic acid accumulation and defense signaling in young and mature *Arabidopsis*

**DOI:** 10.3389/fpls.2014.00775

**Published:** 2015-01-09

**Authors:** Philip Carella, Daniel C. Wilson, Robin K. Cameron

**Affiliations:** Department of Biology, McMaster University, Hamilton, ON, Canada

**Keywords:** *Arabidopsis thaliana*, *Pseudomonas syringae* pv. *tomato*, age-related resistance, salicylic acid, antimicrobial, flowering, senescence, intercellular space

## Abstract

In *Arabidopsis*, much of what we know about the phytohormone salicylic acid (SA) and its role in plant defense comes from experiments using young plants. We are interested in understanding why young plants are susceptible to virulent strains of *Pseudomonas syringae*, while mature plants exhibit a robust defense response known as age-related resistance (ARR). SA-mediated signaling is important for defense in young plants, however, ARR occurs independently of the defense regulators NPR1 and WHY1. Furthermore, intercellular SA accumulation is an important component of ARR, and intercellular washing fluids from ARR-competent plants exhibit antibacterial activity, suggesting that SA acts as an antimicrobial agent in the intercellular space. Young plants accumulate both intracellular and intercellular SA during PAMP- and effector-triggered immunity, however, virulent *P. syringae* promotes susceptibility by suppressing SA accumulation using the phytotoxin coronatine. Here we outline the hypothesis that mature, ARR-competent *Arabidopsis* alleviates coronatine-mediated suppression of SA accumulation. We also explore the role of SA in other mature-plant processes such as flowering and senescence, and discuss their potential impact on ARR.

## INTRODUCTION

The phenolic phytohormone salicylic acid (SA) contributes to a number of developmental and physiological responses in plants. SA is predominately known for its role in initiating defense responses against pathogens such as *Pseudomonas syringae* (reviewed in Vlot et al., 2009; An and Mou, 2011), a hemibiotrophic bacterial pathogen. Seminal research established SA as an essential player in plant defense. Wild-type plants respond to microbial attack by accumulating high levels of SA, which induces expression of PATHOGENESIS-RELATED (PR) proteins, ultimately allowing the plant to respond in a resistant manner (Malamy et al., 1990; Métraux et al., 1990). Importantly, plants with reduced SA levels due to ectopic expression of a bacterial SA-hydroxylase gene (*NahG*) are unable to activate defense responses and are highly susceptible to pathogen attack (Gaffney et al., 1993; Delaney et al., 1994). The level of pathogen-induced SA accumulation is correlated with the magnitude of pathogen resistance, where high levels of SA are associated with resistance and low levels of SA are associated with susceptibility. Thus, SA is a focal point in the tug-of-war between plants and pathogens, with each side attempting to regulate SA levels for its own benefit. Not surprisingly, plant and pathogen genotypes play a large role in dictating the outcome of this tug-of-war, however, an often-overlooked aspect in this struggle is the developmental stage of the plant. In this perspective, we outline the profound impact that developmental age has on SA-mediated plant-pathogen interactions in *Arabidopsis*.

## GENERAL PLANT DEFENSE RESPONSES

Plant defense is comprised of several overlapping layers that include PAMP-triggered immunity (PTI), effector-triggered susceptibility (ETS), and effector-triggered immunity (ETI; reviewed in Jones and Dangl, 2006). Basal defenses such as PTI are induced upon the recognition of common microbial epitopes or PAMPs (pathogen-associated molecular patterns) such as flagellin or chitin by cognate pattern-recognition receptors. The PTI response includes accumulation of SA (reviewed in Boller and Felix, 2009; Meng and Zhang, 2013). SA is synthesized through two distinct metabolic routes. It can be generated from L-phenylalanine via the PAL (PHENYLALANINE AMMONIA LYASE) pathway or from chorismate via ICS1/SID2 (ISOCHORISMATE SYNTHASE1/SALICYLIC ACID INDUCTION DEFICIENT2) the latter of which is responsible for the bulk of chloroplast-derived SA produced during pathogen infection in *Arabidopsis* (reviewed in Vlot et al., 2009; Dempsey et al., 2011). *Arabidopsis sid2* mutants produce little SA and are defective in basal/PTI responses (Nawrath and Métraux, 1999; Wildermuth et al., 2001). To overcome PTI, adapted pathogens employ virulence effector proteins that translocate into plant cells via the type 3 secretion system (T3SS), as well as small diffusible phytotoxins such as coronatine. Once inside the cell, some effector proteins and phytotoxins target host proteins to interfere with PTI, resulting in host susceptibility or enhanced pathogenicity. The mechanisms by which effectors and phytotoxins suppress defense vary, however many suppress plant defenses such as SA accumulation and *PR* gene expression (Xin and He, 2013). To overcome the suppression of plant defense by effector proteins, plants employ ETI. To initiate ETI, an effector protein is first recognized by a highly specific Resistance (R) receptor protein, either directly or indirectly. Recognition of an effector or “avirulence” protein by its cognate R receptor initiates a signaling cascade that results in SA accumulation, *PR* gene expression, and a form of programmed cell death known as the hypersensitive response (Jones and Dangl, 2006). This form of resistance is highly specific and affords a high degree of resistance. Both ETI and PTI also initiate systemic acquired resistance (SAR), a defense response in which an initial local infection induces long-distance signaling to protect distant uninfected leaves against future pathogen attack (reviewed in Champigny and Cameron, 2009; Shah and Zeier, 2013). Much like PTI and ETI, plants defective in SA accumulation are defective in SAR. Although SA itself is not the long-distance SAR signal (Rasmussen et al., 1991; Vernooij et al., 1994), the SA conjugate methyl salicylate (MeSA) participates in SAR (Park et al., 2007; Vlot et al., 2008; Liu et al., 2011).

## MECHANISM OF SA SIGNAL TRANSDUCTION

Salicylic acid accumulation initiates a complex signaling cascade that includes hallmark *PR* gene expression. Early genetic screens for mutants defective in SA signaling discovered NPR1 (NON-EXPRESSOR OF PR1), a transcriptional co-activator important for plant defense (Cao et al., 1997). Our current understanding of SA signaling places NPR1 in a central role as the master-regulator of SA-induced signal transduction (reviewed in Vlot et al., 2009; An and Mou, 2011; Yan and Dong, 2014). In brief, SA accumulation leads to a change in cellular redox status that facilitates the monomerization of a cytosolic oligomer pool of NPR1, which translocate to the nucleus and interact with TGA transcription factors to regulate gene expression (Mou et al., 2003). Although NPR1 plays a central role in signaling, its inability to reliably bind SA in conventional ligand-binding assays suggests that it is not the SA receptor. A search for the SA receptor demonstrated that NPR1 homologs NPR3 and NPR4 bind SA and regulate NPR1 protein stability to mediate SA-signaling (Fu et al., 2012). Based on their findings, the authors depict a model wherein SA levels affect the ability of NPR3 or NPR4 to target NPR1 for ubiquitinylation and degradation via the proteasome. At the lowest and highest levels of SA, the NPR1 homologs direct NPR1 degradation, preventing SA signaling. At intermediate SA levels, NPR1 is no longer targeted for degradation and can participate in SA signaling (reviewed in Yan and Dong, 2014). This regulatory module ensures that SA induces defense gene expression only when necessary and prevents constitutive SA-mediated immune signaling, which is generally detrimental to growth and development (reviewed in Durrant and Dong, 2004; Rivas-San Vicente and Plasencia, 2011).

## MATURITY AND DEFENSE—UNCONVENTIONAL DISEASE RESISTANCE

**FIGURE 1:**
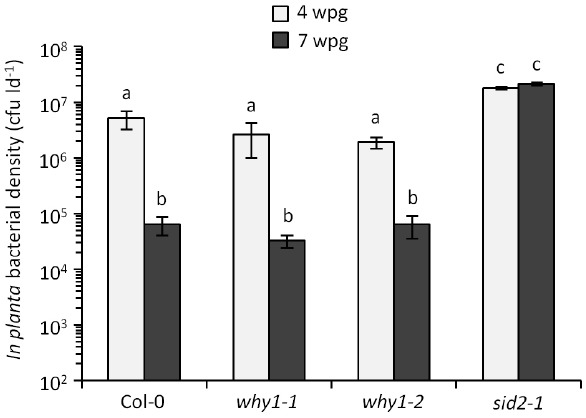
**WHIRLY1 is not required for ARR.** Plants were inoculated with 10^6^ cfu ml^–1^ of virulent *Pst* DC3000 at 4 and 7 weeks post-germination (wpg). *In planta* bacterial density [colony-forming units per leaf disk (cfu ld^–1^)] was determined 3 days later and is presented as the mean ± standard deviation of three sample replicates. Different letters indicate statistically significant differences between means (ANOVA, Tukey’s HSD, *P* < 0.05). This experiment was performed three times with similar results. Plant growth, inoculations, and quantification of bacterial levels were performed as described previously (Carviel et al., 2014). The T-DNA mutants *why1-1* (SALK_023713C) and *why1-2* (SALK_147680C) were obtained from TAIR and have been characterized previously (Isemer et al., 2012).

Much of what we know about SA signaling and its impact on induced resistance comes from experiments using young plants. In the *P. syringae*–*Arabidopsis* pathosystem, young plants inoculated with virulent *P. syringae* pv. *tomato* (*Pst*) support high levels of *in planta* bacterial growth and are susceptible to disease, while mature plants support low levels of *in planta* bacterial growth and are resistant (Kus et al., 2002). This phenomenon, known as age-related resistance (ARR), is a highly robust form of developmentally regulated resistance. The focus of this perspective is ARR in *Arabidopsis*, however, developmentally regulated disease resistance has been observed in a variety of other plants (reviewed in Whalen, 2005; Develey-Rivière and Galiana, 2007). Much like defense in young plants, the ability to accumulate SA in response to pathogen infection is required for ARR in *Arabidopsis*. Plants defective in SA biosynthesis or accumulation (*sid2*, *eds1*, *eds5/sid1*, *NahG*) are ARR-defective such that mature plants remain susceptible to *Pst* at later stages of development (Kus et al., 2002; Carviel et al., 2009, 2014). Unlike defense in young plants, NPR1 is not required for ARR (Kus et al., 2002; Cameron and Zaton, 2004), suggesting that although SA accumulation is critical, NPR1-dependent SA signaling is dispensable during ARR. This led us to speculate that ARR may employ NPR1-independent SA signaling. Our knowledge of NPR1-independent SA signaling is less extensive in comparison to NPR1-dependent responses, however, the ssDNA-binding transcription factor WHIRLY1 (WHY1) is among a small number of genes thought to be involved in NPR1-independent SA signaling and defense (reviewed in Desveaux et al., 2005; An and Mou, 2011). WHY1 is required for SA and pathogen-induced *PR1* expression irrespective of NPR1. Moreover, ssDNA-binding activity of WHY1 is induced by SA treatment in both wild-type and *npr1-1* plants, suggesting that WHY1 functions to induce *PR* expression independent of NPR1 (Desveaux et al., 2004). To investigate the requirement of NPR1-independent SA signaling for ARR, we compared the ARR phenotypes of two independent *why1* T-DNA insertion mutants (*why1-1, why1-2*) to wild-type Col-0 and the SA-deficient *sid2-1* mutant. Plants were inoculated with 10^6^ colony-forming units per ml (cfu ml^–1^) of virulent *Pst* (DC3000) at 4 and 7 weeks post-germination (wpg) followed by determination of *in planta* bacterial density 3 days later (Figure 1). For both wild-type Col-0 and the *why1* mutants, young plants supported high *in planta* bacterial densities (2–5 × 10^6^ cfu per leaf disk [cfu ld^–1^]), whereas mature plants displayed reduced bacterial densities (3–6 × 10^4^ cfu ld^–1^) consistent with a strong ARR response. In comparison, the SA-deficient *sid2-1* mutant displayed a characteristic ARR-defective phenotype, with high *in planta* bacterial densities (>1 × 10^7^ cfu ld^–1^) at 4 and 7 wpg. These data suggest that WHY1 function is not required for ARR. Given that WHY1 and NPR1 are not required for ARR competence, we suggest that SA signaling through these proteins is not an important component of ARR. Indeed, we previously demonstrated that ARR-competent plants express less *PR1* in response to virulent *Pst* compared to young plants (Kus et al., 2002; Rusterucci et al., 2005), indicating that ARR represents an unconventional SA-dependent defense response that occurs in older plants. Although it is possible that SA plays an NPR1- and WHY1-independent signaling role that is not associated with *PR1* expression, we propose that SA may play a different role altogether during ARR.

## A POTENTIAL NON-SIGNALING ROLE FOR SA IN PLANT DEFENSE RESPONSES

An alternative, non-signaling role for SA during ARR was explored by Cameron and Zaton (2004), who hypothesized that SA may act as an antimicrobial agent in the intercellular space (apoplast) during ARR. This hypothesis arose from the observation that intercellular washing fluids (IWFs) collected from mature (ARR-competent) plants inoculated with *Pst* possessed antimicrobial activity that was not present in corresponding IWFs from young (ARR-incompetent) plants (Kus et al., 2002). Moreover, antimicrobial activity was absent in IWFs from mature *NahG* plants, suggesting that SA accumulation is required for the antimicrobial activity observed in mature wild-type plants. Further investigation revealed that SA accumulated in IWFs from mature plants but not young plants following inoculation with *Pst* (Cameron and Zaton, 2004). Infiltration of exogenous SA into the intercellular space rescued the ARR-defect in *sid2-1* but not *NahG*. Conversely, addition of the SA-degrading salicylate hydroxylase enzyme to the intercellular space impaired the ARR response of wild-type plants. Together these data suggest that SA accumulation in the intercellular space is a key aspect of the ARR response. The antimicrobial effect of SA on *Pst in vitro* (Cameron and Zaton, 2004) suggests that SA itself could be acting as an antimicrobial agent *in planta* during ARR. Moreover, SA and structurally related compounds possess antimicrobial activity against a variety of other phytopathogens *in vitro* (Prithiviraj et al., 1997; Georgiou et al., 2000; Amborabé et al., 2002; El-Mougy, 2002; Martín et al., 2010).

Mature plants accumulate high levels of intercellular SA in response to virulent *Pst*, while young plants accumulate relatively little (Cameron and Zaton, 2004; Carviel et al., 2014). We therefore propose that pathogen-mediated suppression of intercellular SA accumulation contributes to disease susceptibility in young plants, and that mature plants are able to overcome this virulence strategy. In young plants the *P. syringae* phytotoxin coronatine has been shown to suppress SA accumulation at the whole-leaf level (deTorresZabala et al., 2009; Zheng et al., 2012) as well as in the intercellular space (Carviel et al., 2014). Young plants inoculated with a coronatine-deficient *Pst* mutant accumulated higher levels of intracellular and intercellular SA, and supported lower bacterial levels compared to plants inoculated with wild-type *Pst* (Carviel et al., 2014). This suggests that intercellular SA accumulation is a component of the basal defense response that is suppressed by *Pst*. A specific signaling pathway for coronatine-mediated suppression of SA accumulation in young plants has recently been uncovered (Zheng et al., 2012), and we hypothesize that ARR involves the activity of one or more developmentally regulated gene products that alleviate coronatine-mediated suppression of defense (Wilson et al., 2014). Similar to mature plants responding to virulent *Pst*, young plants responding to avirulent *Pst* also accumulated high levels of SA in IWFs (Carviel et al., 2014). Thus, intercellular SA accumulation may also contribute to ETI.

## SA-ASSOCIATED MATURE-PLANT PROCESSES AND ARR COMPETENCE

Our ARR research has revealed novel aspects of SA-mediated defense in both young and mature plants. However, the fundamental question, “how do mature plants become competent for ARR?,” remains to be answered. In *Arabidopsis*, several mature-plant developmental processes have been associated with SA accumulation (reviewed in Rivas-San Vicente and Plasencia, 2011). We speculate that these SA-dependent processes may contribute to ARR competence. Below, we briefly describe two major developmental processes, the transition to flowering and leaf senescence, and our efforts to understand their contribution to SA accumulation and ARR.

### IMPACT OF LEAF SENESCENCE AND SA CATABOLISM ON ARR

Leaf senescence is an actively regulated developmental process that coordinates the reallocation of metabolic resources from leaves to reproductive tissues in older plants (reviewed in Lim et al., 2007). As a mature-plant process, leaf senescence could contribute to ARR competence. In a recent study, Zhang et al. (2013) identified the *Arabidopsis* S3H (SA-3-HYDROXYLASE) protein, which is responsible for the catabolism of SA to 2,3-dihydroxybenzoic acid (DHBA) and 2,5-DHBA. *Arabidopsis s3h* mutants accumulated high levels of SA and underwent leaf senescence early, whereas transgenic *Arabidopsis* overexpressing *S3H* had low levels of SA, high levels of 2,3-DHBA sugar conjugates, and were delayed in senescence (Zhang et al., 2013). This study demonstrates the strong positive correlation between SA levels and the induction of leaf senescence. The authors also determined that 2,3-DHBA and its xyloside conjugate 2,3-DHB3X accumulated with age (Zhang et al., 2013). In a previous study, 2,3-DHBA was identified as an EDS1-dependent metabolite that accumulated in response to *P. syringae* infection and with age (Bartsch et al., 2010). Although 2,3-DHBA does not possess a strong capacity to induce *PR1* gene expression, the authors propose that it may contribute to EDS1-dependent defense. We agree with the authors’ idea that 2,3-DHBA, an isochorismate-derived metabolite that accumulates with age and is dependent on EDS1, may contribute to ARR. Their finding that 2,3-DHBA was a poor inducer of *PR1* expression is in agreement with our observations that ARR-competent plants do not express *PR1* to high levels and that ARR doesn’t require NPR1 or WHY1. Whether 2,3-DHBA plays a role in ARR could be addressed by quantifying 2,3-DHBA and 2,3-DHB3X in IWFs collected from young and mature plants inoculated with *Pst*, and by determining if DHBA contributes to the antimicrobial activity of IWFs from ARR-competent plants. However, ARR competence is not associated with early-stage senescence marker gene expression (*SAG-13*) or senescence-induced leaf tip chlorosis (Kus et al., 2002), suggesting that senescence is not a developmental cue for ARR competence. Rather, aspects of leaf aging such as an increase in SA catabolism and DHBA accumulation may contribute to ARR competence in *Arabidopsis* independent of leaf senescence.

### THE TRANSITION TO FLOWERING IS ASSOCIATED WITH ARR

The transition from vegetative to reproductive growth is a highly regulated process that relies on multiple endogenous and environmental cues (reviewed in Amasino, 2010). Interestingly, SA appears to act as a positive regulator of flowering in *Arabidopsis*, as SA-deficient mutants flower later than wild-type plants (Martínez et al., 2004). Detailed genetic analyses indicated that the promotion of flowering by SA appears to proceed through several independent mechanisms, involving components of the autonomous and photoperiod flowering pathways (Martínez et al., 2004). In both short- and long-day-grown *Arabidopsis* the floral transition occurs at approximately the same time as the onset of ARR (Rusterucci et al., 2005). This led us to speculate that the transition to flowering could be a developmental cue for ARR competence. However, further investigation effectively separated the transition to flowering from ARR competence (Wilson et al., 2013). Early-flowering mutants and wild-type plants forced to flower early by transient exposure to long days did not exhibit early ARR, nor did late-flowering mutants display delayed ARR. Together these data suggest that the transition to flowering is neither sufficient nor required for the onset of ARR competence.

Unexpectedly, our analysis of flowering-time mutants revealed that early-flowering *svp-31* was ARR-incompetent. SVP (SHORT VEGETATIVE PHASE) is a MADS-domain transcription factor that acts as a negative regulator of the floral transition (Hartmann et al., 2000). A genome-wide ChIP-chip study (Tao et al., 2012) identified many SVP target genes including three NAC transcription factors that have been shown to mediate the suppression of SA accumulation by coronatine (Zheng et al., 2012). Our current efforts are focused on elucidating the role of SVP in ARR and determining whether SVP suppresses *NAC* gene expression to prevent coronatine-mediated suppression of SA accumulation in mature plants.

## CONCLUSION—DEVELOPMENTAL DIFFERENCES IN SA-MEDIATED DEFENSE

**FIGURE 2:**
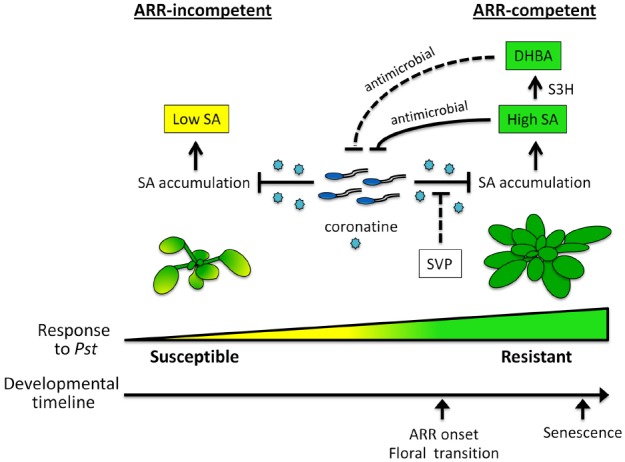
**Salicylic acid-mediated disease resistance in young and mature *Arabidopsis*.** The model illustrates key aspects of the *Arabidopsis* age-related resistance (ARR) response to *Pseudomonas syringae* pv. *tomato (Pst)* with a focus on salicylic acid (SA) accumulation in young and mature plants. In young plants, coronatine produced by *Pst* suppresses the accumulation of SA to dampen defense, resulting in susceptibility to disease. At later stages of development, plants acquire ARR competence and become resistant to *Pst*. Mature plants infected with virulent *Pst* accumulate high levels of SA despite the presence of coronatine. Our accumulated evidence supports the idea that intercellular SA acts as an antimicrobial agent to limit *Pst* growth. The onset of ARR competence coincides with the transition to flowering whereas leaf senescence occurs well after. We hypothesize that the floral repressor SHORT VEGETATIVE PHASE (SVP) contributes to ARR by alleviating coronatine-mediated suppression of SA. SA-3-HYDROXYLASE (S3H) converts SA to 2,3-dihydroxybenzoic acid (DHBA), which accumulates with age and contributes to leaf senescence. We hypothesize that DHBA contributes to ARR as an antimicrobial agent in the intercellular space. Dashed bar—hypothesized relationship, solid bar—relationship supported by evidence.

It is clear that SA plays a central role in immune responses to *Pst* in both young and mature *Arabidopsis*. Moreover, *Arabidopsis* ARR is also effective against the biotrophic pathogen *Hyaloperonospora arabidopsidis* (Hpa; Rusterucci et al., 2005; Carviel et al., 2009). Since several *Hpa* effectors have been shown to suppress SA-mediated immunity in young plants, (Anderson et al., 2012; Caillaud et al., 2013; Asai et al., 2014) we speculate that suppression of SA-mediated defense by *Hpa* is also alleviated in mature ARR-competent plants. Our current model of ARR and the role that SA plays in mature versus young plants is illustrated in Figure 2. At earlier developmental stages, plants support high levels of bacterial growth and are susceptible to *Pst*. The phytotoxin coronatine contributes to the suppression of SA accumulation in young plants to prevent SA-mediated immune signaling, thus promoting disease susceptibility. At later stages of development, plants gain competence for ARR and are resistant to *Pst* infection. This is associated with the accumulation of high levels of SA, which may act as an antimicrobial agent in the intercellular space. The transition to flowering overlaps with the onset of ARR, however, it is not the developmental cue for ARR competence. Interestingly, our recent studies with SVP, a negative regulator of the transition to flowering, suggest that this transcription factor may contribute to ARR by limiting coronatine-mediated suppression of SA accumulation. Further, we hypothesize that the SA-catabolite 2,3-DHBA, acts as an antimicrobial agent in the intercellular space similar to SA. Future research is required to address the key questions posed by our model and clarify the role of SA during plant-pathogen interactions in mature versus young *Arabidopsis*.

### Conflict of Interest Statement

The authors declare that the research was conducted in the absence of any commercial or financial relationships that could be construed as a potential conflict of interest.
